# Breaking out of the citadel: social theory and psychiatry

**DOI:** 10.1192/bjb.2022.17

**Published:** 2023-06

**Authors:** Rob Poole, Catherine A. Robinson

**Affiliations:** 1 Bangor University, UK; 2 University of Manchester, UK

**Keywords:** Philosophy, history of psychiatry, social theory, qualitative research methods, social determinants of mental health

## Abstract

British psychiatry has tended to hold itself aloof from social theory. Nonetheless, these ideas have influenced the development of mental health services. Alongside this, the biopsychosocial model cannot reconcile contradictions in the scientific evidence regarding mental illness. We need to develop a more constructive understanding of the implications of social theory.

From the late 1980s, British psychiatry fell into a long preoccupation with biological concepts and technologies. There was massive investment in genomics and new physical treatments. Biology is always important in psychiatry, but in the years that followed, biological psychiatry showed a weakness for promising more than it delivered. For example, a *Nature Neuroscience* editorial in 1999 stated ‘Conditions such as depression, schizophrenia, stroke and age-related cognitive decline […] are now seen as specific diseases whose causes can be identified and which it will some day be possible to prevent or cure’.^1^

In the 20 years that have followed, the results have been disappointing in terms of truly novel technologies with substantially better treatment outcomes for service users, despite rapid expansion in biological knowledge. Over-enthusiasm about the ‘Decade of the Brain’ skewed emphasis in research funding decisions and educational curricula, and this has had an enduring impact on priorities.

More recently, epidemiological findings have emerged about the importance of childhood adversity and adult social circumstances in mental illness, particularly psychosis.^2–4^ Some of these findings come from research groups investigating biological causal factors, who found that social determinants are as significant as genetics. From a scientific point of view, the biopsychosocial model does not comfortably reconcile apparently contradictory findings from its constituent domains. For example, in the biological domain, there is robust evidence from psychiatric genomic research to suggest that schizophrenia is 70–80% heritable.^5^ That heritability depends on a large number of genes.^6^ On the other hand, in the social domain, there is compelling evidence of a huge increase in risk of being diagnosised with the disorder associated with British Black ethnicity^7^ and, independently, associated with growing up in inner-city deprivation.^8^ It is possible to stretch the stress–vulnerability model to accommodate both sets of findings, but it is scientifically unsatisfactory. The sizes of the effects are so large that the two domains appear to compete for precedence. It is not that findings from one domain are more valid than those from the other. Instead, it is difficult to reconcile the known facts:

‘As a scientific community we find us ourselves in a very awkward place as far as our theoretical models are concerned. There is no generally accepted theoretical stance that can comfortably explain most of what is known. We are rich in information but poor in theoretical understanding.’^9^

## The positivist citadel

Amid growing interest in the social aspects of mental illness, a social history of psychiatry between 1960 and 2010 was published by the Royal College of Psychiatrists in July 2021.^10^ This is a significant re-evaluation of British psychiatry, with major contributions from service users, other disciplines and stakeholders. Our own contribution explores the way that, until recently, organised psychiatry ignored major developments in social theory, often avoiding debate by sheltering in a positivist citadel.^11,12^ This has been unfortunate, as psychiatry is of special interest to social theorists, a broad group of social scientists who seek to understand human activities as meaning-laden social phenomena. Social theory includes much economic and political theory. It is not synonymous with postmodernism, which is just one type of theoretical stance. Some social theorists embrace positivism and, at the other extreme, some reject empricism altogether. Policy makers draw heavily on social theory as a guide to what might be desirable outcomes of policy. Social theorists of the left have been critical of mental healthcare as the exemplar of the sequestration of social deviance, whereas conservative theorists have criticised it as a smothering, dependency-forming threat to liberty.

Psychiatry's failure to properly engage with social theory has not prevented such ideas from influencing policy makers and the non-medical mental health professions, particularly mental health nursing. Through these routes, social theories have had an impact on the development of mental health services, frequently catching psychiatrists unprepared, for example, for changes in the priorities that are set for mental health services and for sharp changes in attitude to the importance of psychiatrists’ role in services. The functionalisation of mental healthcare has reduced it to discrete, time-limited and standardised packages. Interventions are increasingly purchased as packaged commodities from the private sector, neatly meeting neo-liberal socioeconomic aspirations to marketise healthcare.^13^ Continuity of care is incompatible with a so-called ‘recovery model’ that has been developed by services, not by service users. In this model, rapid discharge from services is seen to be synomous with recovery and the worst possible outcome of treatment is dependency, ignoring the reality that some degree of dependency is intrinsic to positive human relationships. Organised psychiatry in the UK is now rightly concerned as it surveys a landscape of atomised care that neglects therapeutic relationships.^14,15^ Services have become difficult to access and increasingly controlling and restrictive of those who meet their criteria.^16^ If we want to change direction towards a more optimistic, more collaborative, less coercive psychiatry with a more coherent scientific basis, we need to engage with social theory.

## Postmodernism

The group of social theories labelled ‘postmodernism’ are generally concerned with processes through which the powerful exert control over the rest of the population.^11^ Laws and policing are held to be only the most explicit elements of this. Social discourse involves patterns of thinking about oneself and the world that are supported by depictions in news media or popular culture and particular linguistic practices, among other influences. A dominant discourse maintains patterns of behaviour and social structures, formal and informal, that serve the interests of powerful social groups. Science itself is seen to have no existence separate from its social function.

In a postmodern critique, the dominant biological discourse in psychiatry of the 1990s is understood to have discharged two functions: the development of financially exploitable technologies such as medications; and the location of emotional distress in brain disorder, rather than in reactions to social adversity. The new selective serotonin reuptake inhibitor (SSRI) antidepressants are thus understood to be both hugely profitable products and a diversion of public understanding of the causes of distress away from social injustice and towards epidemics of illness with no particular social meaning. This ignores psychiatry's role in relieving human suffering, but psychiatry struggles to counter postmodernists’ criticisms of the profession because they clearly have some merit, even if they do not take into account the whole picture. Some prominent mainstream psychiatrists tacitly acknowledge this. For example, Allen Frances, who was Chair of the DSM-IV Task Force and who is by no means an ideological postmodernist, campaigns against the medicalisation of life and the overuse of medications.^17^

Looking back at the impact of the ‘Decade of the Brain’ in the 1990s through the lens of what is now known about Big Pharma on the one hand and the role of urban deprivation in psychosis on the other, open-minded empiricists must acknowledge that some postmodern critiques have some scientific validity. An overemphasis on biological causes and treatments does tend to serve the interests of large corporations and tends to be associated with neglect of service users’ social needs, irrespective of the effectiveness of biological treatment in relieving their symptoms. This acknowledgement does not imply acceptance that mental illness is a myth or that empirical psychiatry should be abolished. If we enter into a dialogue with those social theorists who respect empiricism, it is possible to see that psychiatrists relieve the suffering of many service users while also being part of a discourse that has some adverse sociocultural effects. To take an example, we should be able to acknowledge that while we fight stigma, psychiatry is part of the larger system that generates it. Similarly, empirical knowledge has been shown to be heavily influenced by its socioeconomic context, finding expression in demonstrable distortions of the evidence base, for example through research funding decisions or publication bias.^18^ It is important to understand these ambiguities, and that they do not invalidate the knowledge gained.

## Neo-liberalism

Neo-liberal social theory is equally relevant to modern psychiatry. Thomas Szasz condemned mental illness as a myth and he is often mistakenly lumped in with postmodernist antipsychiatrists. In fact, where postmodernism is a broadly a movement of the left, Szasz was in the same right-wing libertarian tradition as Ayn Rand. Both were unapologetic positivists with similar ideas to the economists who developed neo-liberalism. To Szasz, the only relationship that gives agency to service users and legitimacy to psychiatrists is a paying one, freely entered into by both parties. He saw no grounds to ever deny the responsibility of individuals for their actions and the decisions that they make, no matter how unwell they might seem. Similar reification of choice underlies the rationale for a modern global free market.

Such ideas lead to a doctrine of business management of mental healthcare because it is seen to be a market commodity like any other. To neo-liberals, free will and free choice are central to their conceptualisation of the unrestricted free market as the perfect mechanism to regulate human affairs. In mental health services, this theoretical orientation favours brief manualised or programmatic interventions. Further support for programmatic intervention has come from radical liberals, such as the economist Richard Layard, who reframed the role of government as a duty to maximise happiness. Layard appears to understand mental illness primarily as the opposite of happiness, and one of his major proposals to increase happiness was to maximise accessibility to cognitive–behavioural therapy (CBT).^19^

Layard greatly influenced Tony Blair's government (1997–2007). In our opinion, some of his ideas, including his emphasis on achieving population-level change through intervention at an individual level, would tend to locate him alongside the social theorists of the right. His influence led to a massive expansion of psychological services in England, which was broadly welcomed. Unfortunately, there was a simultaneous, and possibly causal, loss of policy-maker (and research-funder) interest in the needs of people with chronic psychosis.^20^

## Implications of social theory for psychiatry

Psychiatry takes prides in a broad-based scientific approach and in its humanitarian values, but the credibility of its dominant model has been weakened by the replication crisis, a flawed peer review system and recurrent scandals over research fraud.^21^ Highly articulate defenders of psychiatry (most recently, Huda^22^) make arguments that are convincing for those who adhere to a traditional empirical framework. These arguments are much less persuasive to those who do not, because a set of underlying assumptions make it difficult for the profession to address the core concerns of critics even where it accepts that critics have a point (as does Burns in his elegant defence of psychiatry^23^).

Organised psychiatry has become substantially more reflective over the past decade or so. The Royal College of Psychiatrists has apologised for previous abusive practices, such as aversion therapy to change sexual orientation. It has acknowledged the impact of structural racism in mental health services and as a cause of mental disorders. It has been respectful of sincere but flawed attempts to reinvent psychiatry, such as Bracken & Thomas's postmodern psychiatry (‘post-psychiatry’).^24,25^ This progress needs to be consolidated and extended. We need a practical approach to help us reach a less defensive relationship with social science, social theory and public health.

Engagement with social theory need not involve rolling over in the face of every criticism, but we need a more nuanced understanding of our evidence base and of our everyday practice. An important arena is psychiatric journals. Editorial policies need to break free of the hegemony of quantitative findings. We need more space for papers that explore our interactions with service users and with society, including those authorities that fund us. Qualitative research is a powerful empirical tool because it links science to lived experience. It deploys a broad range of social theories and perspectives.^26^ Many medical journals claim to welcome qualitative papers, but few are published. In our experience, there is a dearth of available reviewers with sufficient knowledge to critique them appropriately. We need better representation of the broad range of qualitative reseachers on editorial boards.

The profession is developing a stronger focus on the social determinants of mental health, which itself demands a better understanding of lived experience and an ability to unpack concepts such as social capital,^27^ which are given little attention in our scientific understanding of social adversity. All of these topics, and the social sciences in general, must be properly covered in postgraduate medical education and continuing professional development. They should inform research funding priorities. There are implications for the lack of diversity, especially with regard to social class, in candidate selection criteria for medical schools, postgraduate training schemes and, especially, academic psychiatry. We can and should constructively engage with social theory without betraying psychiatry's core values and strengths.

## Data Availability

Data availability is not applicable to this article as no new data were created or analysed in this study.

## About the authors

**Rob Poole**, MBBS, FRCPsych, is Professor of Social Psychiatry in the Centre for Mental Health and Society, School of Medical and Health Sciences, Bangor University, UK. **Catherine A. Robinson**, BA, PhD, is Professor of Social Care Research, Social Care and Society in the School of Health Sciences, University of Manchester, UK.
